# Surface Plasmon Resonance Sensors on Raman and Fluorescence Spectroscopy

**DOI:** 10.3390/s17122719

**Published:** 2017-11-24

**Authors:** Jiangcai Wang, Weihua Lin, En Cao, Xuefeng Xu, Wenjie Liang, Xiaofang Zhang

**Affiliations:** 1 Beijing Key Laboratory for Magneto-Photoelectrical Composite and Interface Science, School of Mathematics and Physics, University of Science and Technology Beijing, Beijing 100083, China; wjf2534002362@126.com (J.W.); 15501115088@163.com (W.L.); caoen@stu.sdnu.edu.cn (E.C.); xuefengxu8@163.com (X.X.); 2 Beijing National Laboratory for Condensed Matter Physics, Institute of Physics, Chinese Academy of Science, Beijing 100190, China; wjliang@iphy.ac.cn

**Keywords:** plasmon resonance, sensor, nanomaterials, surface plasmon resonance, spectroscopy

## Abstract

The performance of chemical reactions has been enhanced immensely with surface plasmon resonance (SPR)-based sensors. In this review, the principle and application of SPR sensors are introduced and summarized thoroughly. We introduce the mechanism of the SPR sensors and present a thorough summary about the optical design, including the substrate and excitation modes of the surface plasmons. Additionally, the applications based on SPR sensors are described by the Raman and fluorescence spectroscopy in plasmon-driven surface catalytic reactions and the measurement of refractive index sensing, especially.

## 1. Introduction

Surface plasmon resonance (SPR) can significantly enhance the surface sensitivity and accurately detect the whole process of the target molecules’ binding reaction. Based on these advantages, it promptly promotes the advent of SPR sensors, which are powerful tools for real-time supervising of interactions in biology and chemistry analysis [1,2,3,4,5].

Since the first development of SPR in the early 1980s by Nylander and Liedberg [6,7,8], SPR sensing has received sustained attention from the scientific community due to its remarkable performance in characterizing and probing molecular interactions.

To overcome the shortcoming that surface plasmons are difficult to excite, Otto et al. established the configurations of the attenuated total reflection (ATR) method in 1968 [9,10,11,12]. Then, Wood found abnormal diffraction spectra on metal diffraction gratings in 1902, and these abnormal diffraction spectra, proven by Fano, were closely connected with the extinction of electromagnetic surface waves [13,14]. In the 1990s, SPR technology and some optical instruments were combined by Knoll et al. for the development of SPR sensors. Since then, the feasibility of SPR sensors has been broadly proven all over the world [15,16,17,18,19,20,21].

At present, SPR sensors have made significant progress in terms of technology and applications, and researchers are attempting to develop novel techniques and configurations of SPR sensing to overcome the shortcoming of detecting lower molecular weight chemical and biological analysis under extremely dilute conditions. There are several excellent review papers about this [12,14,16,19,22,23]. However, a specific and systematic introduction about the principle of SPR sensors is lacking, as well as the application, especially in the optical surface catalytic reactions and the measurement of refractive index sensing.

Therefore, in this work we focus mainly on the principle and application of SPR sensors combined with previous studies. In principle, how surface plasmons are generated and how they lead to the enhancement of electromagnetic field are introduced in detail. The optical designs are summarized thoroughly, including the type of substrate and the coupled model of the surface plasmon excitation. Moreover, the applications based on SPR sensors are also described in detail, especially in plasmon-driven surface catalytic reactions and the measurement of refractive index sensing, which are revealed by Raman and fluorescence spectroscopy.

## 2. Principle of SPR Sensors

Surface plasmons (SPs) are regarded as the collective coherent oscillations of delocalized electrons, which are stimulated by incident illumination at the interface between a metal and dielectric [24]. The surface plasmon resonance (SPR), created by SPs, can strengthen the localized electromagnetic field tremendously [25,26]. Additionally, SPR is very sensitive to the refractive index attached to the surface of the metal film [27,28,29]. The resonance spectral response of the SPR will change when the conditions of the medium are changed, which can reflect certain properties of the system.

Thus, based on the principles of SPR, SPR sensors are used to detect the molecular information in the enhanced localized electromagnetic field, and are seen as thin-film optical refractometers by measuring the variation of the refractive index on the surface of a metal film. SPR sensors have made significant advances in high-sensitivity spectroscopy and spectral analysis fields.

### 2.1. Mechanisms

The enhanced mechanism of SPR sensors includes electromagnetic enhancement and chemical enhancement. The former is caused by localized SPs, which can enhance the spectra over a large frequency range, and the latter can selectively enhance Raman signals of molecules that are absorbed on the metal surface [30,31,32,33,34,35].

**Figure 1:**
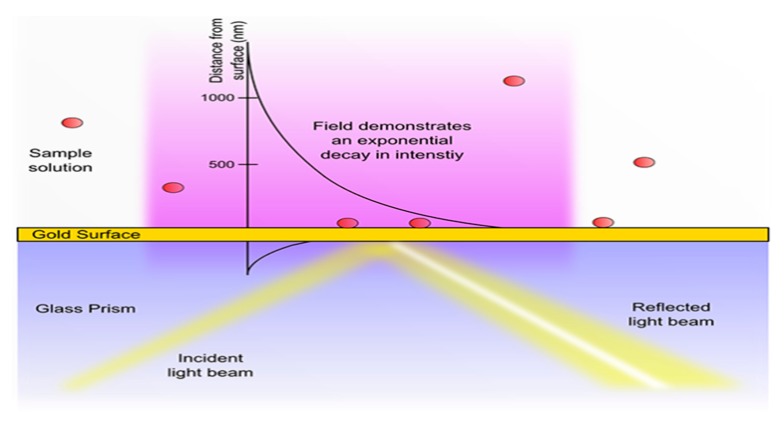
The mechanism of SPR. Referenced from [36]. Reproduced with kind permission from XanTec bioanalytics GmbH.

As mentioned above, the localized SPs play a critical role on SPR sensors, and it can also be seen from the frequency resonance process that the frequency of excited photons is analogous to SPs. In fact, there are many factors that need to be considered when measuring samples. The detailed process is shown in Figure 1.

There is an evanescent field at the sensor surface that does not propagate as an electromagnetic wave, but whose energy is spatially concentrated in the surrounding of oscillating charges which is essential for the initial phase of SPR. When the p-polarized light strikes the interface of the metal film and dielectric medium, the component of the electromagnetic field will penetrate through and create an electromagnetic evanescent field, although photons are reflected directly from the interface (Figure 1). The evanescent wave triggers the free electrons in the metal surface and contributes to the generation of a surface plasmon which causes the enhancement of the electromagnetic field with an exponential decay with the increase in the distance from the surface in the perpendicular direction [36]. Note, with the help of the composite permittivity of the metal and dielectric, the SPs can exist, and according to the Maxwell equation and the boundary condition of the electromagnetic field, the real permittivity of the metal and dielectric medium must be opposite. The real permittivity of the metal must be negative in the visible or near-infrared band, which shows the dispersion relation for the electromagnetic wave, while the imaginary part of permittivity means the absorption of the electromagnetic wave [37,38]. The dispersion relation is described as:

(1) $$\beta_{sp}=\frac{\omega }{c}\sqrt{\frac{\varepsilon_{d}\varepsilon_{m}}{\varepsilon_{d}+\varepsilon_{m}}}=\frac{2\pi }{\lambda }\sqrt{\frac{\varepsilon_{d}\varepsilon_{m}}{\varepsilon_{d}+\varepsilon_{m}}}$$

where *β_sp_* is the propagation constant of the surface plasmon on the interface between the metal and the dielectric; *ω* is the angular frequency; *c* is the speed of light and *λ* is the wavelength of incident light in a vacuum; and $\varepsilon_{m}$ and $\varepsilon_{d}$ represent the permittivity of metal and dielectric, respectively [39].

The propagation constant of the electromagnetic wave is a measurement of the mutative amplitude and phase (which affects the frequency of SPs) when the wave propagates in a given direction. Thus, from the dispersion relation, the SPs must match with the angular frequency and wavelength of incident light. Then, excited SPs are going to have the collective oscillation and greatly enhance the near-field amplitude at the resonance wavelength. The SPs confined to the electromagnetic field carry the corresponding energy and are rapidly converted into high-energy electromagnetic waves, leading to an enormous localized electromagnetic enhancement [23].

### 2.2. Optical Design

SPR sensors are a technique used for detecting molecular information, consisting of SPR technology and some optical modes. The different modes lead to different influences on the surface sensitivity, such as the substrates and coupling mechanism.

#### 2.2.1. Optical Sensors Based on the Excitation of Surface Plasmons

**Figure 2:**
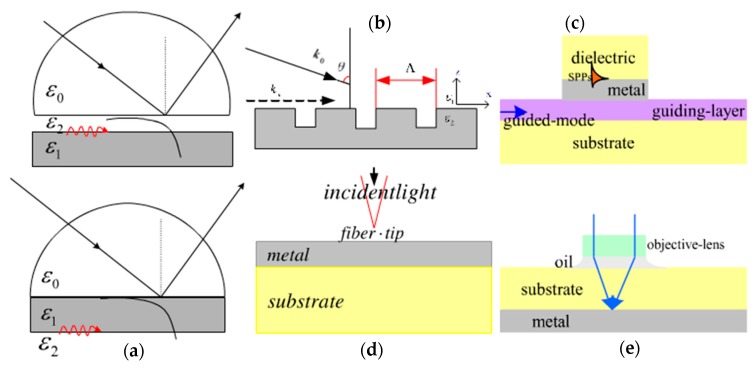
The modes of surface plasmon excitation. (**a**) Prism coupling; (**b**) grating coupling; (**c**) waveguide coupling; (**d**) the near field excitation with the fiber optic probe; (**e**) the high intensity aperture of the objective lens is used to focus the incident beam coupling. Adapted and reorganized from [41].

As we know, the design of optical modes is an important part of SPR sensor systems. The incident laser must be accurately focused onto the interface between the metal and the dielectric, so it is very necessary to choose appropriate coupling modes of SP wave excitation. Normally, a surface plasmon wave can be excited by coupling at the interface between metal and dielectric when the wave vector of the incident light is matched with the propagation constant of the surface plasmons. There are several optical modes designed for surface plasmon waves, as shown in Figure 2.

Prism coupling mode, known as attenuated total reflection (ATR) mode, is the most commonly used in triggering surface plasmons. Figure 2a shows the prism coupling of the two structures: the Otto structure and the Kretschmann structure. In the Otto structure, there is a narrow air gap between the prism and metal, and the full-reflection evanescent wave is used to adjust the wave vector matching condition on the interface between the prism and the air when the incident light irradiates. The SPs can be stimulated at the interface of the metal/dielectric when the following conditions are met. The matching equation condition can be expressed as:

(2) 2πλnpsin(θ)=Re{βSP}

where λ and θ are the wavelength and angle of the incident light, respectively; $n_{p}$ is the refractive index of the prism; and $\beta_{SP}$ represents the propagation constant of the surface plasmon. In the Kretschmann structure, the metal film is directly plated on the prism surface, and the thickness of the metal film is not very thick to excite the SPs wave at the interface of the metal/dielectric. This method is simpler than the Otto method, but may damage the prism [9,40].

Another mode of exciting SPs is grating coupling (Figure 2b): when the light is radiated onto a metallic grating, the diffracted wave will be generated, which can directly couple to SPs if the momentum of the diffracted wave is matched with the grating surface [42]. The matching condition can be defined as:

(3) 2πλndsin(θ)+m2λ∧=±Re(βsp)

where $n_{d}$ is the refractive index of the sensing material; m is an integer and denotes the diffraction order; and ∧ represents the grating period of the metal surface.

Furthermore, waveguide coupling is used to couple to a surface plasmon wave. In waveguide coupling, the evanescent wave can couple the light field energy of the guide mode to the surface plasmons in the guiding layer (Figure 2c), and the stimulated surface plasmon polariton (SPP) wave can propagate and be excited at the outer boundary of the metal surface. The coupling condition is:

(4) βmode=Re{βsp}

where $\beta_{mode}$ represents the propagation constant of the waveguide mode.

The above are the most common three coupling models, and there is another excitation mode based on the fiber optic SPR sensor [43], which uses the core of the optical fiber to replace the prism used in Kretschmann and named “near field excitation with the fiber optic probe” in Figure 2d. In the fiber optic SPR probe, the metal layer is coated on the unclad core of the fiber and the sample kept around the metal layer is sensed. There are some types of geometry regarding the fiber optic SPR sensor [44,45,46]. For example, two fibers with different core diameters were connected by thermal fusion splicing to leak the transmitted power into the cladding layer of the small core diameter fiber so that the leaked light may induce an evanescent wave required for the excitation of surface plasmons [47]. In strongly-focused beam coupling, the microscopic objective lens with a high numerical aperture is near the metal substrate through the oil layer. The incident light passes the microscopic objective lens and focuses on the dielectric substrate/metal interface to achieve SPP wave excitation [7,48] (Figure 2e).

In these optical mode coupling processes, the energy of the incident light will transform into SPs and spread on the surface of the metal. The light wave exciting the SPs has a change in phase, due to the change in light intensity [39].

#### 2.2.2. Substrates

**Figure 3:**
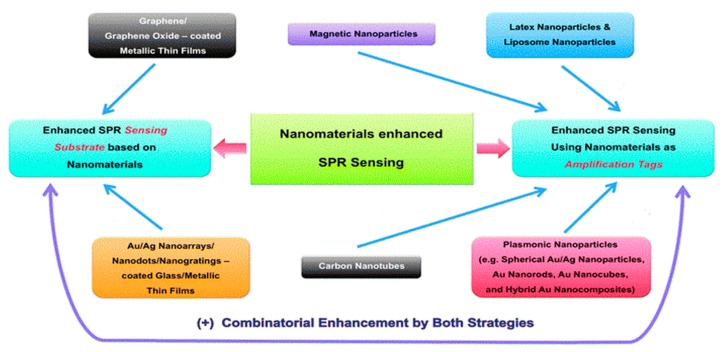
Various types of nanomaterials for enhanced SPR sensing. Reproduced from ref. [49] with permission of The Royal Society of Chemistry.

Noble metal, such as Au and Ag, servedas a suitable candidate for better optical response and electric field enhancement in the visible and near-infrared light bands. Additionally, the size and morphology of the metal also play a significant role on regulating the enhancement of SPR sensing signals. The noble metallic nanoparticles are often used to improve the performance of sensing. There are several nanomaterials applied in SPR sensors, as displayed in Figure 3.

Actually, there are two main forms, named “substrates” and “amplification tags”, also called “plasmonic nanoparticles”, which are widely applied in SPR sensors, as shown in Figure 3. The different plasmonic nanoparticles contribute differently to the enhancement of SPR sensing.

Over the past few decades, the Au/Ag nanoparticles were widely applied in the enhancement of SPR sensors, but there are some shortcomings, such as the high cost, and instability which leads to easy oxidation in air [50,51,52]. Thus, the researchers have to explore other plasmonic nanoparticles to enhance the signal of SPR sensing. Magnetic nanoparticles (MNPs) have been employed for amplifying the signal of SPR sensors [53,54,55]. The MNPs can be controlled by external magnetic fields, and develop into an “aggregate” layer which has a strong refractive index contrast on the metallic film of the sensor. This layer will reveal a remarkable SPR signal when measuring samples [56]. Compared to conventional plasmonic nanoparticles, the MNPs are lower cost and have higher sensitivity. Additionally, graphene-modified SPR sensors have also attracted much attention, which coat a graphene layer on the metal (Ag or Ag) thin films. This leads to the significant enhancement of the excited electric field due to the superconductivity of graphene, which greatly promotes the charge transfer to the metal thin films. It can also effectively prevent oxidation of silver when coating graphene layers on Ag thin film [57,58,59,60,61]. Hence, the MNPs and graphene-modified SPR sensors are currently the most popular SPR sensors. In addition, molecular-imprinted polymers (MIPs) [62] aggregated by the imprint molecule (target molecule) based on synthetic polymers, is a promising candidate to be applied in SPR sensors due to their outstanding stability and relatively low cost. More important reasons are that the higher affinity and stronger selectivity are reflected by binding sites of creation that have access to the antibody-antigen system during imprinting. Based on the advantage that they can combine selectively with the target, MIPs have been applied extensively in many study aspects, such as biological synthesis and catalysis, and immunology [63,64]. However, there are still some challenges to be considered, such as the compatibility of MIPs’ binding sites with water’s homogeneity and the synthesis of MIPs with specificity to protein [65]. These are the new trends of further study for MIPs. Latex nanoparticles, liposome nanoparticles, and various metal nanostructures are also used extensively, with a detailed introduction in [66,67,68].

## 3. Types of SPR Sensors

Usually, SPR sensors have two types, including localized surface plasmon resonance (LSPR) sensors and propagating surface plasmon polariton (PSPP) sensors [69].

**Figure 4:**
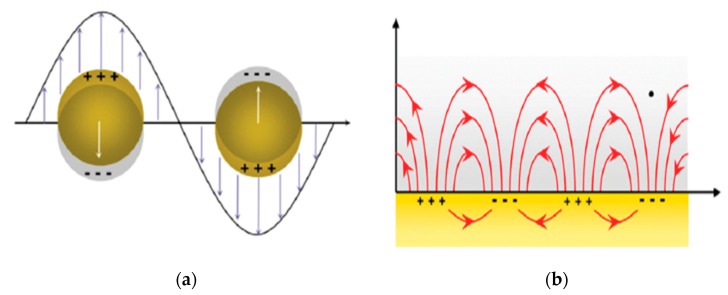
SPR sensors mechanism. (**a**) The mechanism of the LSPR sensor; (**b**) the mechanism of the PSPP sensor. Reprinted from [68] with the permission from Elsevier.

The former is that LSPs are limited in a nanoparticle, where the size is analogous to the wavelength of light used to excite the plasmons is even smaller, or is confined to the gap between metal nanoparticles and which can produce larger electromagnetic fields than the nanoparticles. The LSPs restricted to the surface of metal nanoparticles are density oscillations of charge when the laser excites the surface of the Au or Ag nanostructures (Figure 4a) [70], and they can generate electronic oscillations whose form is an exponential attenuation in the vertical direction (Figure 1). Consequently, this leads to the strong localized electromagnetic field and the significant extinction at the plasmon resonant frequency, even breaking the diffraction limit of light when the energy of the electromagnetic field is aggregated to a scale that is much smaller than the wavelength of the incident light [71,72,73]. It is noteworthy that the enhanced electromagnetic field will greatly promote surface catalytic reactions, and the plasmon resonant frequency can be acquired from the extinction peaks (LSPR peaks) which rely highly on the refractive index of the environment.

In the other, propagating surface plasmon polaritons (PSPPs) based on the surface plasmon polaritons (SPPs), are typically excited by the coupling between SPs and photons on the metal film, and the wave of SPPs can propagate hundreds of micrometers along the interface of the metal-dielectric in the electromagnetic field region [74]. This has a unique advantage in that the incident laser can avoid direct exposure of the measured sample, which avoids interference and damage of the strong background noise and high energy laser on the measured sample (Figure 4b).

## 4. Applications

Numerous SPR sensors have been developed for detection and characterization of molecular interactions in chemistry and biology, such as environmental protection, food safety, and medical care [75,76,77,78,79,80,81,82]. Here, we will provide a review of sensor-based SPR in surface catalytic reactions [83,84,85], especially in localized surface plasmon resonance, and the measurement of the refractive index. The performance is revealed by Raman and fluorescence spectroscopy.

Raman spectroscopy is a spectroscopic technique to detect the vibration, rotation, and other low-frequency modes of some specific molecules or functional groups in a system [86]. Essentially, when the molecule is irradiated by the incident laser in the visible, near-infrared, or near-ultraviolet ranges, the laser will interact with vibration of the molecule and scattering will occur, resulting in the energy of the laser photons being shifted up or down. Hence, Raman spectroscopy is dependent on the principle of inelastic scattering and we can clearly speculate the information of measuring molecules by the shift in energy. In fact, spontaneous Raman scattering is typically very weak, so, in practical applications, we must be supported with some tools or means which divide the Raman spectrum into many types, including surface-enhanced Raman, resonance Raman, tip-enhanced Raman spectroscopy and so on [83,87,88,89,90,91].

Additionally, there is no real transition of the energy level because the laser does not excite the molecule. The Raman Effect is based on the interaction between the electron cloud of a sample and the external electrical field of the monochromatic light [92]. This phenomenon should be distinguished completely from fluorescence spectroscopy in that photons emitted by the molecule from the excited electronic state returns to the ground state under different incident laser events.

Fluorescence spectroscopy is primarily concerned with the electronic and vibrational states. In fluorescence, the molecule must be excited by the incident laser, firstly, and reach an excited electronic state by absorbing photons. Then it will emit photons at different frequencies from the excited state to the ground state, as molecules return to different vibrational levels of the ground state [93]. Therefore, we can obtain the different vibrational structure of the molecule by analyzing the different frequencies of light emitted in fluorescent spectroscopy.

In a word, Raman spectroscopy and fluorescence spectroscopy can detect precisely the information of the molecular structure and can test, in real-time, the changes of the system by enhancing the surface sensitivity.

### 4.1. Plasmon-Driven Surface Catalytic Reactions

Several discoveries have been demonstrated that local surface plasmon resonance (LSPR) can harvest electromagnetic energy and significantly promote surface catalytic reactions because it provides extremely strongly-confined electromagnetic energy and hot electrons [94,95].

When the ‘hot’ electrons scatter into the excited state, the ‘plasmon-driven reaction’ will occur by decreasing the activation energy [91,96,97]. Surface-catalyzed reactions have been investigated since 2010 [5,98], and plasmon-driven irreversible chemical reactions were also demonstrated in an aqueous environment [85,99]. These studies focus mainly on the application of local surface plasmons and are revealed by surface-enhanced Raman scattering (SERS) spectroscopy. SERS is a surface-sensitive technique that enhances Raman scattering of molecules adsorbed on rough metal surfaces or nanostructures [100,101,102], which can contribute to explicitly observing the Raman imaging in spectral analysis [103,104]. Thus, it reveals the significant enhancement in the sensitivity of Raman spectroscopy.

Based on the principle of LSPR and SERS technology, the plasmon-driven reactions can overcome the limitation of the low Raman scattering cross-section by optical diffraction, and detect molecular information by SERS spectroscopy. The following data shows some Raman peaks are selectively enhanced, theoretically analyzed by Sun et al. [5,101,102].

**Figure 5:**
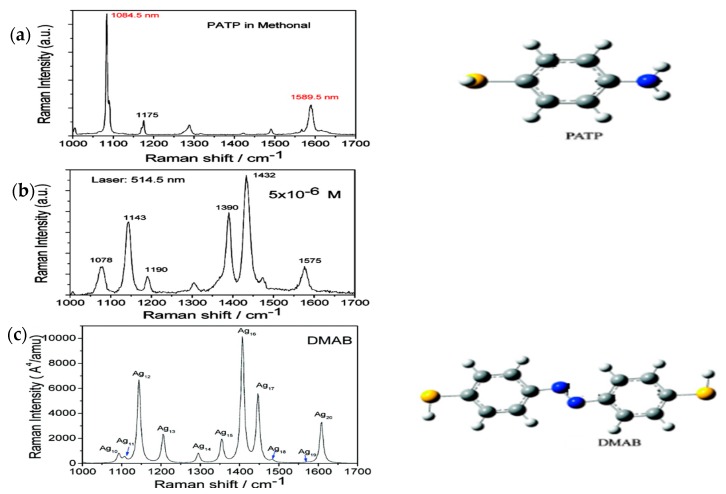
(**a**) Experimental of normal spectra of PATP; (**b**) the experimental of SERS spectra of PATP in a Ag sol; and (**c**) the stimulated Raman spectrum of DMAB. The chemical structures of PATP and DNAB are shown on the right side. Reprinted with the permission from [5]. Copyright 2010 American Chemical Society.

SERS of 4-aminothiophenol (PATP) absorbed on a metal surface have been discussed and measured in extensive studies [105,106,107,108,109,110,111,112,113], and there are three strongly-enhanced Raman peaks (1143 cm^−1^, 1390 cm^−1^, and 1432 cm^−1^) that were once described as a chemical mechanism by Osawa in 1994 [114]. However, such an enhanced phenomenon was not convincingly recognized until 2009, in when experimental SERS spectra of PATP was shown to be analogous to simulated spectra. In 2010, p,p′-dimercaptoazobenzene (DMAB) was theoretically predicted and experimentally shown to be produced from PATP on the surface-catalyzed reaction, assisted by local SPs (Figure 5). Figure 5 reveals the concrete process of DMAB generated from PATP. We can clearly see that there are only two sharp Raman peaks in the normal Raman scattering spectrum of PATP (1084.5 cm^−1^ and 1589.5 cm^−1^) (Figure 5a). However, this remarkable scene took place when PATP iwass measured on Ag nanoparticles, where three peaks were significantly enhanced at 1143 cm^−1^, 1390 cm^−1^, and 1432 cm^−1^ (Figure 5b). This scene is evidenced perfectly in Figure 5c, describing the simulated Raman spectroscopy of DMAB. Based on this phenomenon, and molecular structures of PATP and DMAB (Figure 5), the assumption that PATP is dimerized to DMAB by a surface-catalyzed reaction can be established.

**Figure 6:**
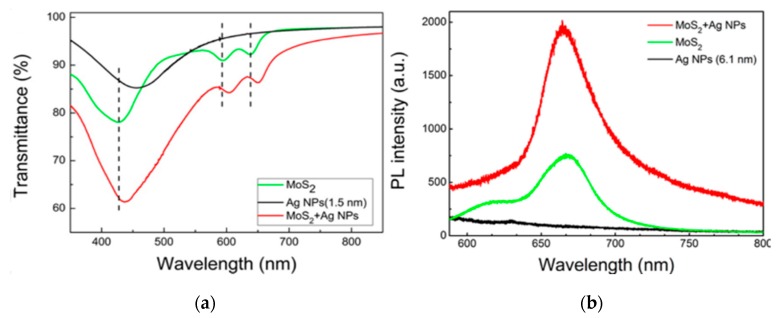
(**a**) The transmission spectra of Ag NPs (1.5 nm), monolayer MoS_2_ and MoS_2_/Ag NPs hybrids on quartz substrates; (**b**) the photoluminescence (PL) spectrum of MoS_2_ enhanced by local surface plasmon resonance. Adapted from ref. [123] with the permission from Elsevier.

According to these analysis results, we know that SERS, based on LSPR, improves the sensitivity by making some unexpected Raman peaks undergo very large selective enhancement in the plasmon-driven reaction. Likewise, this effect also occurs in plasmon-exciton coupling reactions, revealed by transmission and photoluminescence (PL) spectra, as shown in Figure 6.

The monolayer MoS_2_, which has the novel optical property of high transparency and catalytic capabilities, is a promising candidate for inducing surface catalytic reactions when combined with Ag nanostructures [115,116]. We can obviously observe that there is an unexpected change in the transmission spectra for the plasmon-exciton couplings of monolayer MoS_2_/Ag nanoparticle (NP) hybrids (Figure 6a). In other words, the plasmon-exciton couplings of monolayer MoS_2_/Ag NP hybrids has better absorption than only monolayer MoS_2_ and Ag NPs, even more than twice the absorption rate. Similarly, the photoluminescence (PL) spectrum of MoS_2_/Ag NP hybrids is also significantly enhanced due to local surface plasmon resonance (Figure 6b). The emergence of these phenomena mainly the result of plasmon-exciton coupling reactions.

As we know, an exciton is a combination state of an electron and an electron hole, which exists in insulators, semiconductors, and in some liquids. The exciton can be generated when a photon is absorbed by a semiconductor (here, MoS_2_), and excites the surface plasmon hot electrons from the valence band into the conduction band quickly. In the conduction band, the electron is effectively attracted to the localized hole by the repulsive Coulomb forces that are produced by large numbers of electrons surrounding the hole and excited electron. In this process, ultrafast plasmonic hot electrons efficiently fill the holes from electron-hole excitons. It transfers thermal energy to the surface of MoS_2_, and the additional high-energy electrons (hot carriers) on the surface of MoS_2_ and the plasmonic electromagnetic energy can efficiently co-drive surface catalytic reactions. These are the specific processes that can effectively create surface catalytic reactions by the coupling between the plasmon and exciton.

The plasmon-driven surface catalytic reactions based on LSPR can also be applied in other chemical reactions of dependent factors [117,118,119], such as temperature-dependent sensors and pH-dependent sensors. Additionally, the remote excitation of surface plasmon-enhanced Raman scattering (RE-SERS) has also been introduced into plasmonic catalysis in which propagating SPPs behaved as carriers for catalysis and sensing, and a further detailed introduction is described in [120,121,122].

### 4.2. Measurement of Refractive Index Sensing

**Figure 7:**
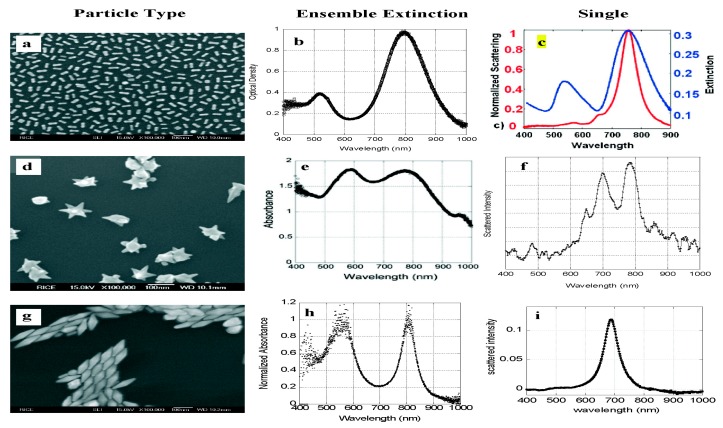
Comparison of the ensemble extinction and single-particle scattering spectra of three particle types; (**a**) gold nanorods; (**b**) nanorod ensemble extinction; (**c**) the extinction and scattering spectra of single-particle; (**d**) Gold nanostars; (**e**) nanostar ensemble extinction; (**f**) nanostar single-particle scattering spectrum; (**g**) gold bipyramids; (**h**) bipyramid ensemble extinction; and (**i**) bipyramid single-particle scattering spectrum. Reprinted with the permission from [124]. Copyright 2010 American Chemical Society.

As mentioned above, SPR sensors are thin-film optical refractometers which measure the shifts of the LSPR peak wavelength. Usually, LSPR peaks are obtained by spectral extinction measurements on the film and spectral scattering measurements on single nanoparticles [124]. Figure 7 shows the difference of these two measurements, revealed by the spectra of three nanoparticle shapes.

Clearly, for small gold nanorods, there are two strong peaks in the ensemble extinction of nanoparticles, but the scattering of the single nonoparticle is too weak to be observed (Figure 7b,c). This means absorption dominates the extinction. For gold nanostars, we can clearly see the peaks of the extinction greatly depends on the fine structure of molecules. Individual nanostars have strong scattering spectra with multiple divided peaks which correspond to resonances of the arms of the nanostars, and the ensemble extinction just has the broad peaks which overlap the small peaks (Figure 7e,f). Thus, it is obvious to distinguish the measurement between ensemble and single-particles by the scattering spectrum [125,126]. For gold bipyramids, due to the spheres and the molecule of the bipyramids, the ensemble extinction has two peaks. However, when the scattering spectrum of a single particle is considered, there will only be one sharp and strong peak, which is different from the spectral wavelength of the ensemble extinction [127,128,129] (Figure 7h,i).

According to the analysis above, the measuring methods need to be suitably selected when the LSPR peak wavelength of the sample is not determined. After the LSPR peaks are measured on Au nanoparticles, the refractive index that clearly reflects the optical properties of the tested sample can be obviously observed by the wavelength of LSPR peak shift. This phenomenon is also equally well revealed by Ag nanoparticles and Pd nanoparticles.

**Figure 8:**
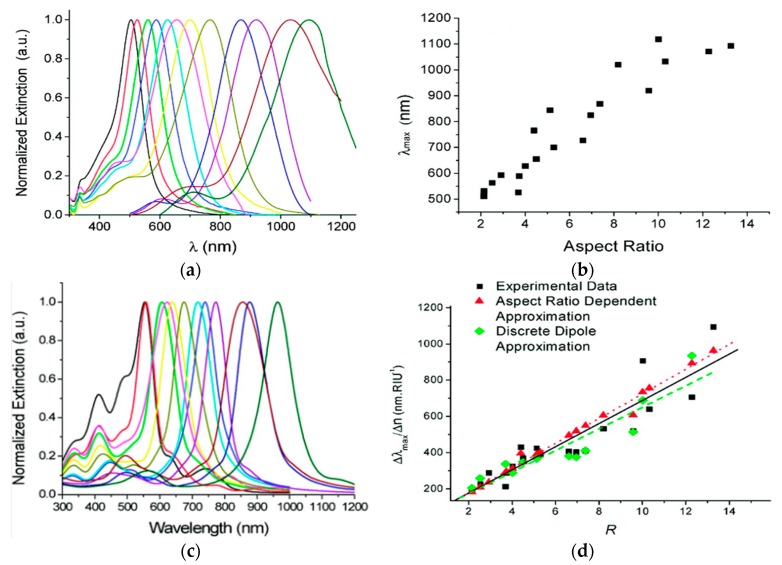
(**a**) Tunability of the LSPR peaks of the TSNP sols within the visible and NIR; (**b**) plot depicting the dependence of the peak wavelength of ensembles on the mean aspect ratio measured for the various samples; (**c**) DDA calculated spectra for TSNP of various aspect ratios; and (**d**) the dependence of the linear refractive index sensitivity on the aspect ratio. Adapted with the permission from ref. [130]. Copyright 2010 American Chemical Society.

Undoubtedly, most of the previous studies are accustomed to the use of gold nanoparticles instead of silver in SPR sensors. The reason not only due to the advantages of gold but, more importantly, silver has a significant drawback in that it is high oxidized in air. To overcome this shortcoming, solution phase triangular silver nanoplates (TSNP) appeared which have been applied in altering the surface chemistry, as well as the tunable localized surface plasmon refractive index sensors with the potential of highly-responsive bio-sensing [130]. Unlike the previously-studied solution of silver nanoplates, the stabilizing polymer can be ignored during preparation and has a narrow geometric distribution, and the TSNP sols contribute to the sample fabrication for sensitivity analysis and have a highly-consistent response to the interaction with the electromagnetic field. Here, LSPR bulk refractive index sensitivities were clearly observed with LSPR peaks throughout the UV-NIR wavelength region and various aspect ratios (Figure 8).

The LSPR extinction spectra of TSNP were measured under different edge lengths (Figure 8a), which show the strong resonance at different LSPR wavelengths and obviously display the redshift within the range of 500–1150 nm, especially. The ensembles’ peaks wavelength at the aspect ratios from two to 13 are comprehensively studied (Figure 8b), showing the same trend in redshift as the aspect ratio increases and has an approximated linear relationship. To further evaluate the potential application of TSNP sols for various refractive indices of the surroundings, especially the specific relationship of LSPR peaks varying with the refractive index around the nanoplates, the bulk refractive index of TSNP is changed in different sucrose concentrations. In the case of various aspect ratios and other unchanged parameters, the specific relationship between the sensitivity of LSPR peaks and the change of the refractive index are demonstrated. Figure 8c shows the extinction spectra of TSNP under different aspect ratios, which are calculated by the discrete dipole approximation (DDA), which is a numerical method of solving Maxwell’s equations for absorption, extinction, and scattering cross-sections of a single nanoparticle [62], and the LSPR peaks are also obviously found to redshift with the increasing aspect ratio. Meanwhile, the refractive index sensitivity shows the linear fit with the increase of the aspect ratio up to 800 nm (Figure 8d). These phenomena are in agreement with the experimental results and prove the tight dependency of LSPR peaks and medium refractive index changes. Additionally, these also highlight that TSNP sols are shown to be ighly-sensitive local surface plasmon refractive index sensors.

A new plasmon resonance effect of a single silver nanocube was also observed, in which appeared two distinct peaks when the silver nanocube was located on the glass substrate in dark-field microscopy. Compared with the LSPR scattering peaks and the refractive index sensitivity of single nanocubes in different dielectric environments, the new plasmon resonance effect have a higher figure of merit for chemical sensing applications and exceptional sensing capabilities. These results also demonstrate, powerfully, the close dependency of the LSPR peak on the refractive index. A more detailed description can be found in [131].

**Figure 9:**
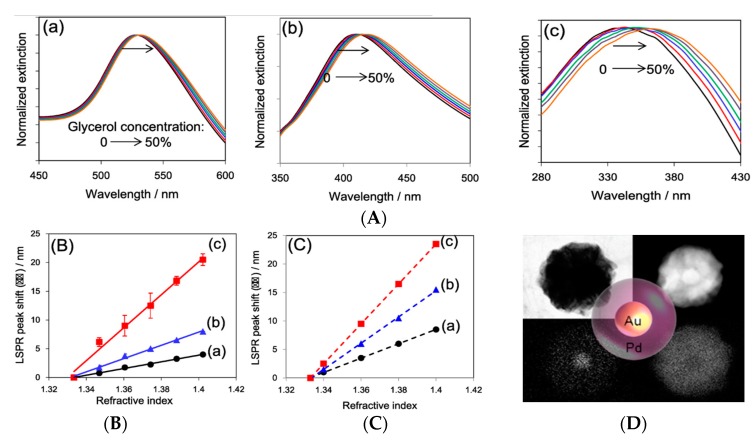
(**A**) Extinction spectra in different solvent mixtures, which are supported by the substrate of the Au NSs (**a**), Ag NSs (**b**), and Au/Pd NSs (**c**); (**B**) experimentally-obtained dependence of the LSPR peak shifts on RI changes for Au NSs (**a**), Ag NSs (**b**), and Au/Pd NSs (**c**); (**C**) the calculated dependence of the LSPR peak shifts on RI changes for AuNSs (**a**), AgNSs (**b**), and PdNSs (**c**) under the same conditions as (**B**); and (**D**) the morphology of Au/Pd NSs are obtained by high-angle annular dark-field scanning transmission electron microscopy (HAADF-STEM). Adapted with the permission from ref. [132]. Copyright 2010 American Chemical Society.

Additionally, Pd nanospheres (Pd NSs) have been applied in a high-sensitivity LSPR sensor recently, which exhibits much higher susceptibility of the LSPR peaks to medium refractive index changes than commonly-used plasmonic sensing materials, such as Au and Ag [132]. The specific sensitivity of the response to refractive index changes are shown in Figure 9. Here, the measurement of Au/Pd NSs in a core–shell structure and morphological information are shown in Figure 9D, and are used as a substrate and compared with Au NSs and Ag NSs in different solvent mixtures (0, 10, 20, 30, 40, and 50 vol% glycerol aqueous solutions). There is such a phenomenon that all the LSPR peaks of the nanospheres will produce a redshift with an increase in the glycerol concentration, especially for Au/Pd NSs, which shift obviously (Figure 9A). That is to say, Au/Pd NSs have a higher sensitivity for the refractive index change of the surrounding medium and this conclusion is perfectly validated in Figure 9B,C, which was revealed experimentally and calculated by the dependence of the LSPR peak shifts on the refractive index changes. This study also powerfully demonstrates that the Pd nanoparticles will be a potential candidate for “the third plasmonic sensing material” to be used in ultrahigh-sensitivity LSPR sensors.

From the above we can see that the refractive index of the surrounding medium has a linear relationship with the LSPR peaks. Similarly, the plasmonic nanoparticles will lead to the redshifts of the LSPR peaks with an increase of the refractive index of the surrounding medium in the plasmon extinction band. In order to clearly introduce the relationship of the refractive index and wavelength of the LSPR peak, we explore the formula step-by-step. First, $\varepsilon_{1}$, which is the dielectric function of medium, needs to be introduced in the Drude model:

(5) $$\varepsilon_{1}=1-\frac{\omega_{p}^{2}}{\omega^{2}+\gamma^{2}}$$

where $\omega_{p}$ is the frequency of plasmon and γ is the damping parameter of the bulk metal. Due to $\gamma <<\omega_{p}$ the visible and near-infrared light bands can be described as:

(6) $$\varepsilon_{1}=1-\frac{{\omega_{p}}^{2}}{\omega^{2}}$$

Combined with the resonance condition ($\varepsilon_{1}=-2\varepsilon_{m}$) and Equation (3), we can obtain the LSPR peak frequency ($\omega_{\max}$):

(7) $$\omega_{\max}=\frac{\omega_{p}}{\sqrt{2\varepsilon_{m}+1}}$$

Then, according to the conversion, including frequency and wavelength ($\lambda =\frac{2\pi c}{\omega }$), the dielectric constant, and the refractive index ($\varepsilon_{m}=n^{2}$), the above can be finally expressed as:

(8) $$\lambda_{\max}=\lambda_{p}\sqrt{2{n_{m}}^{2}+1}$$

where $\lambda_{\max}$ is the wavelength of the LSPR peak and $\lambda_{p}$ is the plasmon wavelength of the bulk metal; $n_{m}$ is the refractive index of metal film surface.

From Equation (8), we can obviously see that the refractive index depends closely on the wavelength of the LSPR peak and can be detected and calculated in time. There is no doubt that the measurement of the refractive index based on SRP sensors will significantly improve understanding of the study, especially in biosensor sensing [23]. It has been demonstrated that the sensitivity of the SPR sensors is closely related to the refractive index, and a detailed introduction is revealed in [23,124].

## 5. Summary

In this overview, the mechanism and excitation modes of surface plasmons attached to SPR sensors are introduced in detail. Based on the principle of SPR, SPR sensors with localized surface plasmons and propagating surface plasmon polaritons can excite strong surface plasmons to induce surface chemical reactions and measure variations in the refractive index on the surface of a metal film by testing spectroscopy. To be specific, surface plasmons limited between nanoparticles can create electromagnetic energy and hot electrons, significantly promoting surface catalytic reactions and change the wavelength of the extinction peak, which greatly affects the refractive index. Our review can provide an understanding of the physical mechanism system of SPR sensors and applications in optical surface catalytic fields for ultrasensitive spectral analysis technology.
